# Synthesis and Antibacterial Evaluation of Chlorhexidine- and Triclosan-Impregnated Kaolinite Nanocomposites

**DOI:** 10.3390/ma18010174

**Published:** 2025-01-03

**Authors:** Aruzhan Alimbek, Zhanar Bekissanova, Bayansulu Otegenova, Ardak Jumagaziyeva, Bagashar B. Zhaksybay, Yana Zhumadilova, Alyiya Ospanova

**Affiliations:** 1 Faculty of Chemistry and Chemical Technology, Al-Farabi Kazakh National University, Almaty 050040, Kazakhstan; alimbek_aruzhan1@live.kaznu.kz (A.A.); zhanar.bekissanova@kaznu.edu.kz (Z.B.); otegenova_bayansulu1@live.kaznu.kz (B.O.); bagazhaksybay@gmail.com (B.B.Z.); 2 Center of Physical-Chemical Methods of Research and Analysis, Almaty 050012, Kazakhstan; 3 Scientific Center for Anti-Infectious Drugs, Almaty 050060, Kazakhstan; r_dawa@mail.ru; 4 Department of Chemistry, Nazarbayev University, Astana 010000, Kazakhstan; yana.zhumadilova@nu.edu.kz

**Keywords:** kaolinite, composite, kaolinite/chlorhexidine, kaolinite/triclosan antibacterial activity

## Abstract

Clay minerals are actively used to obtain a bioactive composite. Kaolinite, as a representative of clay minerals, possesses unique properties essential for the creation of biocomposite materials. This mineral, characterized by its distinctive layered structure, is chemically inert, highly stable, thermally resistant, eco-friendly, biocompatible, and non-toxic. Kaolinite, which plays the role of a carrier in this work, has such properties and can be the basis for biologically active composites. Antibacterial composites, namely, kaolinite/chlorhexidine and kaolinite/triclosan, were synthesized by impregnation of calcined and non-calcined samples of natural kaolinite with the antibacterial agents chlorhexidine and triclosan. The structure, morphology, elemental composition, and mineralogical characteristics of the natural and synthesized kaolinite/chlorhexidine (KAO/CHX) and kaolinite/triclosan (KAO/TCS) composites were investigated by methods of analysis such as X-ray diffraction, FTIR (Fourier-transform infrared) spectroscopy, and scanning electron microscopy. The calcined kaolinite/chlorhexidine composite at 500 °C (KAO_500°C_/CHX) exhibited a higher content of antiseptics compared to the non-calcined kaolinite composite. The antibacterial activities of the kaolinite/chlorhexidine and kaolinite/triclosan composites were investigated against Gram-positive *Staphylococcus epidermidis* and Gram-negative *Klebsiella pneumoniae* and *Escherichia coli* strains by the well diffusion method and dilution method. The highest zone of inhibition was observed against Staphylococcus epidermidis (30.00 ± 0.00 mm and 30.67 ± 0.58 mm) by applying KAO/TCS and KAO_500°C_/TCS via the well diffusion method. The minimum bactericidal concentration of the kaolinite/TCS composite was 15.63 μg/mL for *Staphylococcus epidermidis* and *Klebsiella pneumoniae.*

## 1. Introduction

Currently, one of the cutting edge areas of medical research is the development of materials that effectively prevent microbial growth [1]. In this regard, clay minerals, serving as inorganic carriers, have garnered increased attention [2]. Clay materials act as inorganic ordered bulk fillers [3,4]. These fillers can be in the form of very finely ground materials having an average particle diameter of less than 0.1 μm [5], and their specific surface area is typically above 10 m^2^/g [6] and in the range of 20–300 m^2^/g [7,8]. Examples of such fillers include calcium carbonate [9], diatomaceous silica [10], and various types of clay and alumina (hydrated or non-hydrated) [11]. The filler may have a hydrophobic surface, which is obtained by treating the filler with, for example, suitable silanes, short-chain siloxanes, fatty acids, or resinous silicone materials according to Khansinee Longkaew [12]. The special properties of clay fillers include the presence of adsorption centers on which it is possible to apply any substances with specific properties for the intended purpose, such as active agents for medical purposes [13]. Typically, these agents are selected from antibacterial agents such as chlorhexidine [14], triclosan, silver and its derivatives, hemostatic agents, analgesic agents, etc. [15]. Combining the properties of the clay materials with those of the bioactive agents allows to obtain composites with desired characteristics [16,17].

Clay minerals are key ingredients in anti-inflammatory, antibacterial, and wound-healing products due to their advanced functional properties [18]. Clay minerals play a role in a wide range of dermocosmetic products and various cosmetics, such as liquid [19] and powder foundations, as active dermatologic ingredients [20] and as a styptic agent for commercial wound-dressing products [21]. Most of the important properties attributed to clays for dermocosmetic applications are related to their surface properties (surface area, cation exchange capacity, layer charge, etc.); rheological properties (thixotrophicity, rheopecticity, viscosity, plasticity) [22,23]; and other physical and mechanical properties, including particle size and shape, color, softness, opacity, reflectivity, iridescence, etc. [24].

The nanocomposites based on kaolinite and natural kaolinite modified with various antibacterial agents commonly used in industry and are known for their availability, cost effectiveness, and environmental friendliness [25]. The use of kaolinite in modified drug delivery and the modulation and control of immune response for the treatment of various diseases is a new frontier in the use of kaolinite in biomedicine [26].

Various studies on the preparation of kaolinite-based composite materials are widespread. Kaolinite, as a type of natural clay, has a negative surface charge and therefore can be a suitable carrier for positively charged particles or metal ions [27]. For example, inorganic species with antibacterial properties such as silver ions [28], copper ions [29,30], and zinc ions [31] can be added to kaolinite. In addition to inorganic species with antibacterial properties, organic antibacterial agents such as cetylpyridinium bromide (CPB) [32] and chlorhexidine acetate [1,33] can be added to kaolinite. The combination of two or more antibacterial agents immobilized on kaolinite further enhances the antibacterial activity of the composite due to a synergistic effect [34]. Seow Khai Jou et al. [35] investigated a composite based on silver-modified kaolinite loaded with chlorhexidine that displayed high antibacterial activity against a wide range of bacteria. In addition, the obtained composite based on kaolinite intercalated with urea and treated with chlorhexidine diacetate was found to be a long-acting antibacterial material [36].

The preparation of samples of clay minerals, including kaolin, provides a preliminary elimination of the many impurities of the accompanying metal ions. Shuli Ding et al. [37] explained that the acid and alkaline heat treatment of samples additionally increases the porosity of kaolinite and hence the number of active centers of adsorption and the enhancement of the electrostatic interaction with the desired reagents. Therefore, both natural and modified kaolinite can potentially be a support for many drugs, and composites based on them can be used in medicine.

The aim of this research was to produce an antibacterial composite based on natural, calcined kaolinite and promising antiseptics chlorhexidine and triclosan and study its antibacterial activity against Gram-positive *Staphylococcus epidermidis* and Gram-negative pathogens *Klebsiella pneumoniae* and *Escherichia coli*. Kaolinite-based composites have practical applications in the production of medical and biological materials, including wound-healing products, ointments, and antibacterial hemostatic agents. The conditions of the formation of antibacterial composites based on domestic kaolinite has not been previously investigated by other authors, although Kazakhstan has large reserves of kaolinite, the processing of which is economically and environmentally beneficial.

## 2. Materials and Methods

### 2.1. The Process of Preparation of the Chlorhexidine/Kaolin, Triclosan/Kaolin Composites

The natural kaolinite [26] was obtained from the Alekseev clay deposit, Kokshetau region of the Republic of Kazakhstan (53°30′54″ N, 69°30′40″ E). Samples of natural kaolinite (KAO) were dissolved in water and separated to obtain a granulometric fraction of particle size d_p_ < 0.050 mm. Then, samples were dried at 100 °C.

(1) KAO_500_ is a natural kaolinite sample that was pre-washed with distilled water, dried at 100 °C, and then calcined for 5 h in a muffle furnace at 500 °C.
(2) KAO/CHX is a composite based on natural kaolinite dried at 100 °C and impregnated with the antibacterial agent chlorhexidine digluconate (CHX).
(3) KAO_500_/CHX is a composite based on natural kaolinite dried at 100 °C and calcined at 500 °C for 5 h and impregnated with the antibacterial agent chlorhexidine digluconate (CHX).
(4) KAO/TCS is a composite based on natural kaolinite dried at 100 °C and impregnated with the antibacterial agent triclosan.
(5) KAO_500_/TCS is a composite based on natural kaolinite dried at 100 °C and calcined at 500 °C and impregnated with the antibacterial agent triclosan.

Kaolinite samples were pre-washed with distilled water and dried in an oven at 100 °C, and heat treatment was carried out for 5 h in a muffle furnace at 500 °C (KAO_500_). The composites based on natural, calcined kaolin were obtained via impregnation with antibacterial agents: chlorhexidine (KAO_500_/CHX) and triclosan (KAO_500_/TCS). An amount of 1 g of calcined and uncalcined kaolinite was impregnated with 50 mL of 0.05% chlorhexidine digluconate (CHX, Sigma Aldrich, St. Louis, MO 63103, USA, solution 20%) and triclosan (Triclosan 97% active substance, TCS, Sigma Aldrich, St. Louis, MO 63103, USA) for 5 h. Then, the liquid phase was separated from the solid phase and dried in a desiccator for 4 h at 80 °C.

Natural kaolinite is a light gray substance, soft to the touch; calcined at 500 °C, kaolinite samples become white and visually different from each other. All quantitative characteristics are presented as the mean ± SD using GraphPad Prism version 8.01 (*p* < 0.05).

**Figure 1:**
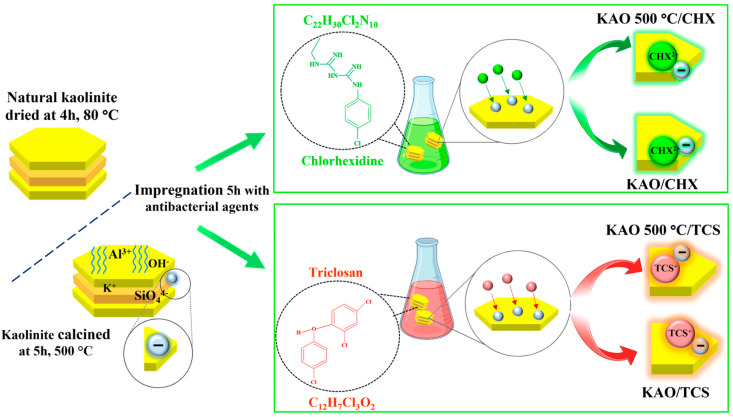
Scheme of the mechanism of the modification of the antibacterial agents chlorhexidine and triclosan on the surface of the natural kaolinite.

The scheme of the mechanism of modification of the antibacterial agents chlorhexidine and triclosan on the surface of natural kaolinite is presented in Figure 1.

### 2.2. Methods and Instrumental Characterization

The morphology and elemental composition of the natural kaolinite and obtained kaolinite/chlorhexidine and kaolinite/triclosan composites were studied by scanning electron microscopy in combination with energy dispersive X-ray (EDX) analysis (SEM-EDX Quanta 3D 200i Dual system, FEI, Houston, TX, USA). The chemical and structural changes in the natural kaolin and obtained kaolinite/chlorhexidine and kaolinite/triclosan composites were studied by FTIR spectrometry (Shimadzu IR Prestige-21/FTIR-8400S FTIR spectrometry, KBr tablets, resolution 4.0, Kyoto, Japan). The mineral composition of the natural kaolinite and obtained kaolinite/chlorhexidine and kaolinite/triclosan composites were characterized by the X-ray powder diffraction (XRD) method (XRD; Rigaku, MiniFlex 600, Tokyo, Japan) using Cu-Kα radiation at 40 kV, 15 mA. Measurements were performed over a 2θ range from 3° to 100° at a rate of 2°/min and a step of 0.01°. The PDF-2 database was used for transcription. The specific surface area was determined by the BET method (Sorbtometer-M 220 V, 50 Hz, P = 250 W). The grain size of natural kaolinite was determined by optical laser diffraction method implemented on an ANALYSETTE 22 NeXT Nano particle analyzer (Fritsch, Berlin, Germany). For this purpose, a sample of natural kaolinite was dispersed in distilled water under the action of an ultrasonic bath until a stable suspension was obtained. The number of measurements was at least 3 for each sample. The result was presented in the form of particle size distribution diagrams according to their weight contribution, and the mean values were determined. The dispersion stability of natural kaolinite represented by zeta potential values was ascertained using a Zetasizer Nano Series instrument (Malvern Instruments, Malvern, UK). The samples of natural kaolinite (at a concentration of 0.05 mg/mL) were dissolved in water with a pH of 2.03, 3.01, 5.04, 8.02, 10.02, and 12.00 and sonicated for 360 min at 25 °C. A Folded Capillary cell (DTS1070) was then used for measurement. Three replicates were used in the measurements of a natural kaolinite sample.

### 2.3. Antimicrobial Assay of Kaolinite/Chlorhexidine and Kaolinite/Triclosan Composites

The antimicrobial activity of the obtained kaolinite/chlorhexidine and kaolinite/triclosan composites was investigated by the well diffusion method in dense Mueller–Hinton agar. Mueller–Hinton agar (MHA) is recommended by EUCAST and CLSI for disc diffusion antimicrobial susceptibility testing (AST). As test strains, one Gram-positive (*Staphylococcus epidermidis* ATCC 12228) and two Gram-negative (*Klebsiella pneumoniae* ATCC 10031, *Escherichia coli* ATCC 8739) strains of bacteria were selected. The selection of these bacteria as test strains is justified due to their prevalence in clinical practice and diversity of pathogenic occurrence making them excellent candidates for evaluating the effectiveness of antimicrobial agents. All test strains were obtained from the American Type Culture Collection (ATCC). The stock inoculum for each test strain was prepared by the direct colony method: an aliquot of the test strain was taken with a bacteriological loop, transferred to a test tube with a sterile saline, and thoroughly homogenized until a homogeneous suspension was obtained. The density of the suspension of each studied strain was 0.5 McFarland, confirmed spectrophotometrically (DEN-1, Biosan, Riga, Latvia), which corresponds to ~1.5 × 10^8^ CFU/mL. The Petri dishes with Mueller–Hinton agar were inoculated with the tested suspension. Sterile cotton swabs were used for inoculation; they were immersed in the bacterial suspension, then slightly squeezed against the walls of the test tube, shaded in three directions, turning the Petri dish by 60°. Then, 6 mm diameter wells were made in the agar, and 200 μL of each sample was added to the respective wells. The plates were placed at room temperature for 30 min to let the samples diffuse into the agar.

The plates were incubated at 37 ± 1 °C in an incubator for 24 h to examine the zone of inhibition. All the experiments were carried out in triplicate, and the results were expressed as the mean ± standard deviation using Excel. The antibacterial activity was determined by measuring the diameter of the inhibition zones in millimeters (mm) around wells using an antibiotic zone scale. The study of the antibacterial properties of the composites was carried out in vitro; however, in the future, it is planned to carry out in vivo studies for the promising composites related to cytotoxicity and pre-clinical tests.

## 3. Results

### 3.1. Characterization of Natural Kaolinite

**Figure 2:**
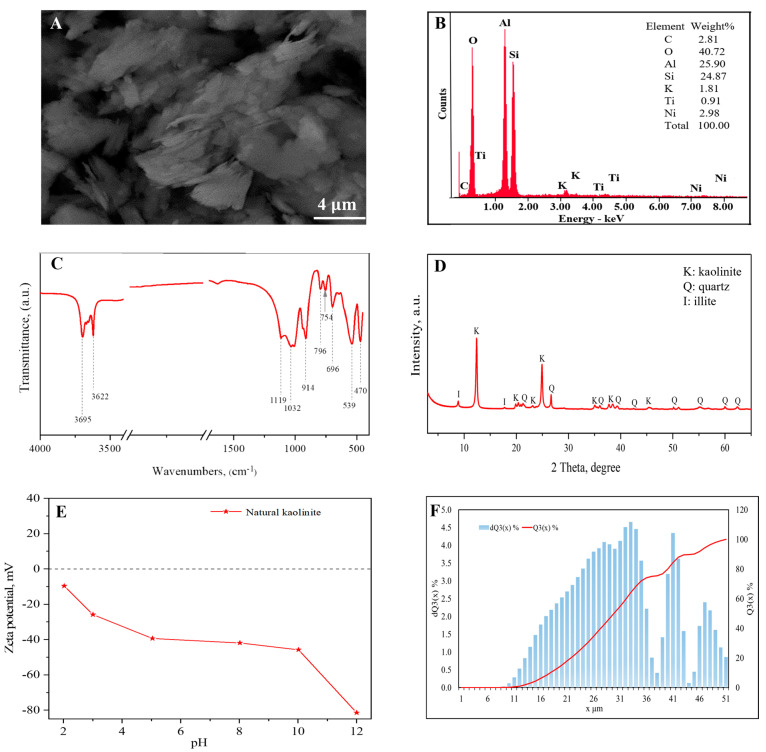
SEM image (**A**), SEM-EDX spectrum (**B**), FTIR spectrum (**C**), XRD patterns (K-kaolinite, Q-quartz, I-illite) (**D**), (**E**) zeta potential value, and (**F**) particle size distribution of natural kaolinite.

The structure and morphology of natural kaolinite were characterized by SEM investigations (Figure 2A). Kaolinite represents a layered structure consisting of two basal (001) planes: a tetrahedral silica sheet in which O atoms are bonded to Si atoms, called the “siloxane surface”, and an octahedral alumina sheet in which OH groups are bonded to Al, called the “aluminol surface”. Both sheets share apical atoms of O [25]. Stacked pseudo-hexagonal platelets with a regular booklet-like shape of kaolinite are observed. Also, fragments of irregularly distributed laminated plate-like particles are present. The elemental analysis (Figure 2B) showed that natural kaolinite consists mainly of oxygen, silicon, and aluminum and contains minor amounts of potassium, titanium, and nickel. The FTIR spectra of kaolinite in Figure 2C show bands at 3695 cm^−1^ and 3622 cm^−1^, which correspond to a hydroxyl group (-OH) in the inner surface [38,39]. In addition, the peaks at 796 and 539 cm^−1^ are assigned to the vibration bands of Si-O-Al bonds, and the peak at 470 cm^−1^ corresponds to the vibration band of Si-O [38]. The band at 914 indicate the presence of kaolinite [39]. Functionalized kaolin groups for Si-O stretching vibrations are seen at 1119 cm^−1^ [40,41]. The peak at 914 cm^−1^ is attributed to the bending vibrations of Al-OH groups on the kaolin surface. The band at 1032 cm^−1^ is attributed to the tensile vibrations of Si-O-Si and Al-O-Al bonds [42]. According to the X-ray diffraction analysis (Figure 2D), natural kaolin consists of kaolinite with small admixtures of illite and quartz. The characteristic peaks of kaolinite in the kaolin structure appear at 12.39°, 20.39°, 24.91°, 35.47°, 38.44°, 45.43°, and 47.98° (ref.code: ICSD_027713), while the peaks of illite and quartz are observed at 26.67° (ref.code: COD_9013718) and 20.89° (ref.code: ICSD_031228), respectively, based on the 2 theta measurements. Figure 2E shows the effect of pH on the zeta potential of natural kaolinite. At a pH range from 2 to 12, the zeta value (ζ) of the potential of the materials ranges from −3.94 to −81.47 mV. A negative value shows the existence of negatively charged functional groups on the kaolinite surface, probably through the dissociation of hydroxyl groups [43]. The granulometric analysis of incremental particle size fraction dQ3(x) % and cumulative size distribution Q3(x) % of the particle size of natural kaolinite are represented in Figure 2F. The range of particle size was between 1 and 51 μm. The main fraction of about 80% of particles were concentrated in the range of about 15 to 35 μm. That indicated the predominance of medium-sized particles in this range.

### 3.2. SEM and SEM-EDX Analysis of Kaolinite/Chlorhexidine and Kaolinite/Triclosan Composites

**Figure 3:**
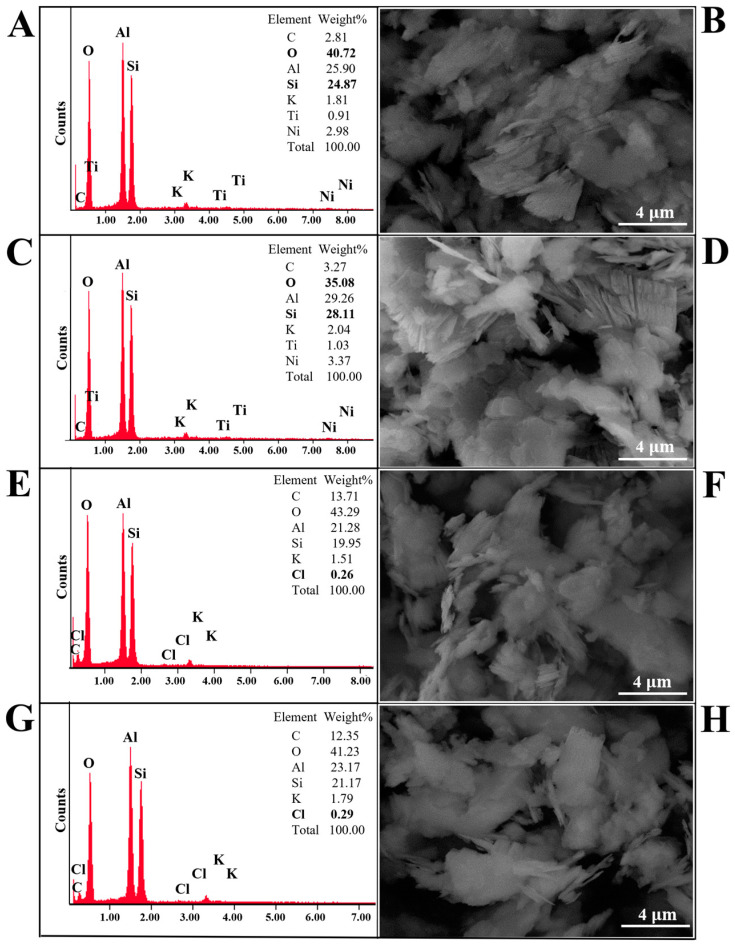
SEM images and SEM-EDX spectra of (**A**,**B**) KAO, (**C**,**D**) KAO_500_, (**E**,**F**) KAO/CHX, and (**G**,**H**) KAO_500_/CHX.

**Figure 4:**
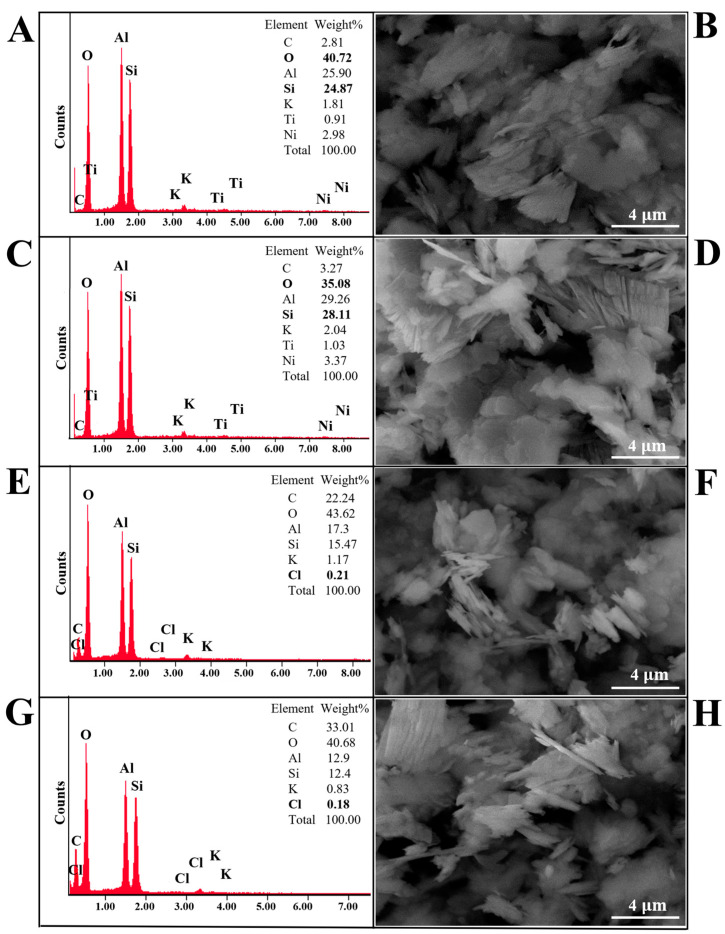
SEM images and SEM-EDX spectra of (**A**,**B**) KAO, (**C**,**D**) KAO_500_, (**E**,**F**) KAO/TCS, and (**G**,**H**) KAO_500_/TCS.

The morphology, structure, and elemental composition of natural kaolinite, calcined natural kaolinite, and the obtained kaolinite/chlorhexidine and kaolinite/triclosan composites are shown in Figure 3 and Figure 4. It was determined that there were no visible morphological changes in the structure of natural kaolinite and the obtained kaolinite/chlorhexidine and kaolinite/triclosan composites. Plate-like structures with multiple layers were observed in all samples. After the washing of natural kaolin, many metal ions are transferred to the solution, and some metals such as titanium and nickel are no longer present in the treated kaolin. The results of the elemental (EDX) analysis showed that the natural kaolinite and obtained composites contain chemical elements such as oxygen, silicon, and aluminum and a minor amount of potassium. The obtained composites were also shown to contain chlorine. It can be concluded that the impregnation of the samples with chlorhexidine and triclosan caused chemical changes in the composition of kaolinite, as shown in Figure 3 and Figure 4E–H. It was also observed that the KAO_500_/CHX and KAO_500_/TCS samples (Figure 3 and Figure 4G,H, respectively) had a higher chlorine content than the KAO/CHX and KAO/TCS samples (Figure 3 and Figure 4E,F). This indicates that the thermal treatment of kaolinite led to the occurrence of transformations, the consequence of which, among others, is an increase in the specific surface area and average grain size of the particles [44]. It can be concluded that this contributes to the better adsorption of antibacterial agents on the kaolinite surface and higher chlorine content.

When developing the conditions for obtaining antibacterial composites based on kaolin, the specific surface area of natural kaolin was studied, which amounted to 16.03 ± 0.75 m^2^/g. For calcined kaolinite, at 500 °C, it changed to 18.43 ± 0.96 m^2^/g. The authors in [45] indicated that the mechanism of the formation of composites based on natural clay montmorillonite and chitosan is due to the purely electrostatic interaction of the cationic polymer with the negatively charged active sites of the clay. The authors in [35] indicated that chlorhexidine cations can react with open basal hydroxy groups or are adsorbed on the negatively charged crystal edge of kaolinite through ionic bonding. This process occurs because at a physiologic pH, chlorhexidine salts can dissociate and release a positively charged chlorhexidine cation [46]. Ralph Grim [47] discussed that the negative surface charge of kaolinite is mainly formed by broken edge bonds and open basal hydroxy groups. In contrast to the broken bonds, the open surface hydroxy groups can be substituted by exchangeable cations such as chlorhexidine [48]. Also, positively charged triclosan ions can be adsorbed on the kaolinite surface. This may occur through the attraction of opposite charges between the kaolinite surface and the triclosan. These interaction mechanisms allow triclosan to penetrate the kaolinite structure and be retained on the surface or within the material. Based on all this, it was assumed that the obtaining of the kaolinite/chlorhexidine and kaolinite/triclosan composites was due to the attraction of opposite charges between the kaolinite surface and the antibacterial agents.

The only difference between natural and modified kaolin is that during the calcination of kaolin at 500 °C, the specific surface area of kaolin increases from 16.03 to 18.43 m^2^/g, and the existing water molecules and OH groups are removed during heat treatment, and positively charged centers appear in the kaolinite structure in addition to a negative charge at the sites of the removal of the OH groups [44,49]. Therefore, calcined kaolinite combines with more CHX and TCS as the chlorine ions of both antiseptics are attracted more strongly than in the case of natural kaolinite, and the results of the elemental analysis indicate an increase in chlorine in the composites with calcined kaolinite (Figure 3 and Figure 4).

Kaolinite is commonly recognized for its negatively charged surface. The authors [47] concluded that the negative surface charge of kaolinite is mainly formed by broken edge bonds and open basal hydroxy groups. Unlike the broken bonds, the open surface hydroxy groups can be substituted by exchangeable cations [48]. At a physiologic pH, chlorhexidine salts can dissociate and release a positively charged chlorhexidine cation [46]. Chlorhexidine cations can react with open basal hydroxy groups or are adsorbed on the negatively charged crystal edge of kaolinite through ionic bonding [35]. Also, positively charged triclosan ions can be adsorbed on the kaolinite surface. This may occur through the attraction of opposite charges between the kaolinite surface and the triclosan. These interaction mechanisms allow triclosan to penetrate the kaolinite structure and be retained on the surface or within the material.

### 3.3. FTIR Analysis of Kaolinite/Chlorhexidine and Kaolinite/Triclosan Composites

**Figure 5:**
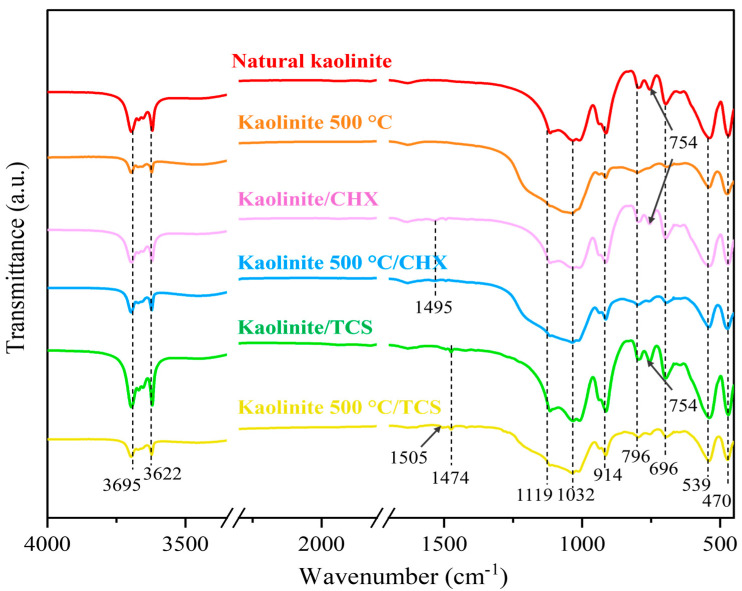
FTIR spectra of KAO, KAO_500_, KAO/CHX, KAO_500_/CHX, KAO/TCS, and KAO_500_/TCS.

The functional groups in the composites were characterized by the FTIR method. According to the FTIR results (Figure 5), the same bands are observed in all the samples, both in natural kaolinite and the obtained composites.

The resulting band at 1032 cm^−1^ is due to the alternating stretching vibrations of Si-O-Si and Al-O-Al bonds [50]. The characteristic oscillation at 796 cm^−1^ refers to Me-OH-Me (Me = Fe, Mg, Al) bonds [42]. The peak at 539 cm^−1^ corresponds to fluctuations in Al-O-Si bonds, where Al is present in an octahedral coordination [42]. The wide bands at 3695 cm^−1^ are due to tensile fluctuations in the OH groups (Al-OH-Al) located at the edges of the kaolin surface [45]. The peak at 914 cm^−1^ is associated with the bending vibrations of the Al-OH groups on the surface of the kaolinite [46].

From the literature, it is known that the bands at 2860 and 2930 cm^−1^ refer to the symmetric and asymmetric valence vibrations, respectively, of the methylene group of chlorhexidine acetate [51]. These bands are weakly expressed on the IR spectrum. The band at 1500 cm^−1^ is associated with C=C stretching from the aromatic ring, which is associated with chlorhexidine [52]. Characteristic absorption bands at 1505 cm^−1^ are observed, which correspond to the C-C stretching of the benzene ring of triclosan [53]. The C-H bending vibrations of aliphatic chains around 1474 cm^−1^ and 1495 cm^−1^ are associated with other vibrations of triclosan. In triclosan, they correspond to specific functional groups.

### 3.4. Powder X-Ray Diffraction Analysis of Chlorhexidine/Kaolin and Triclosan/Kaolin Composites

**Figure 6:**
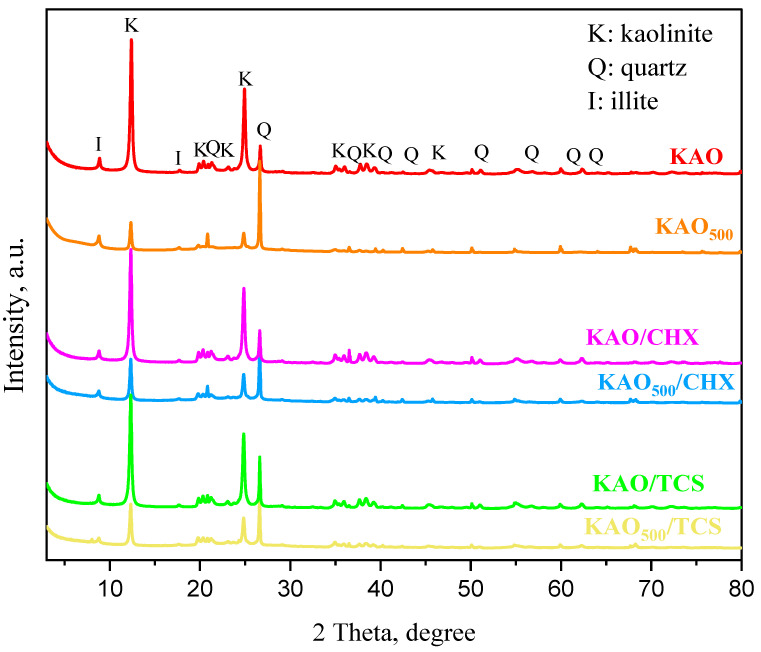
X-ray diffraction patterns of KAO, KAO_500_, KAO/CHX, KAO_500_/CHX, KAO/TCS, and KAO_500_/TCS.

The X-ray diffraction analysis (Figure 6) revealed that natural kaolinite is primarily composed of kaolinite, with minor impurities of illite and quartz. The characteristic kaolinite peaks in the kaolin structure are observed at 12.39°, 20.39°, 24.91°, 35.47°, 38.44°, 45.43°, and 47.98° (ref. code: ICSD_027713). The peaks corresponding to illite and quartz are detected at 26.67° (ref. code: COD_9013718) and 20.89° (ref. code: ICSD_031228), respectively, as determined by the 2θ measurements. When a comparison of the X-ray patterns of the natural kaolinite and kaolinite samples with antibacterial agents was made, no significant changes in the X-ray patterns and theta values were found, and all characteristic reflections were in the same positions. This means that the adsorption of the antibacterial agents occurs on the surface of kaolin rather than intercalating between the layers. According to [54], the intercalation of guest species molecules into kaolinite interlayers obviously increased the interlayer space.

In addition, it was observed that in the KAO_500_/CHX and KAO_500_/TCS samples, some peaks lost their intensity. This is associated with the dehydroxylation process during the calcination of kaolinite, which results in the formation of a new phase called metakaolinite [55]. It can be concluded that the dehydroxylation process leads to the disruption of the structure due to the breakage of unstable bonds and, consequently, to the loss of high intensity peaks in the samples exposed to thermal treatment.

### 3.5. Antibacterial Activity

**Figure 7:**
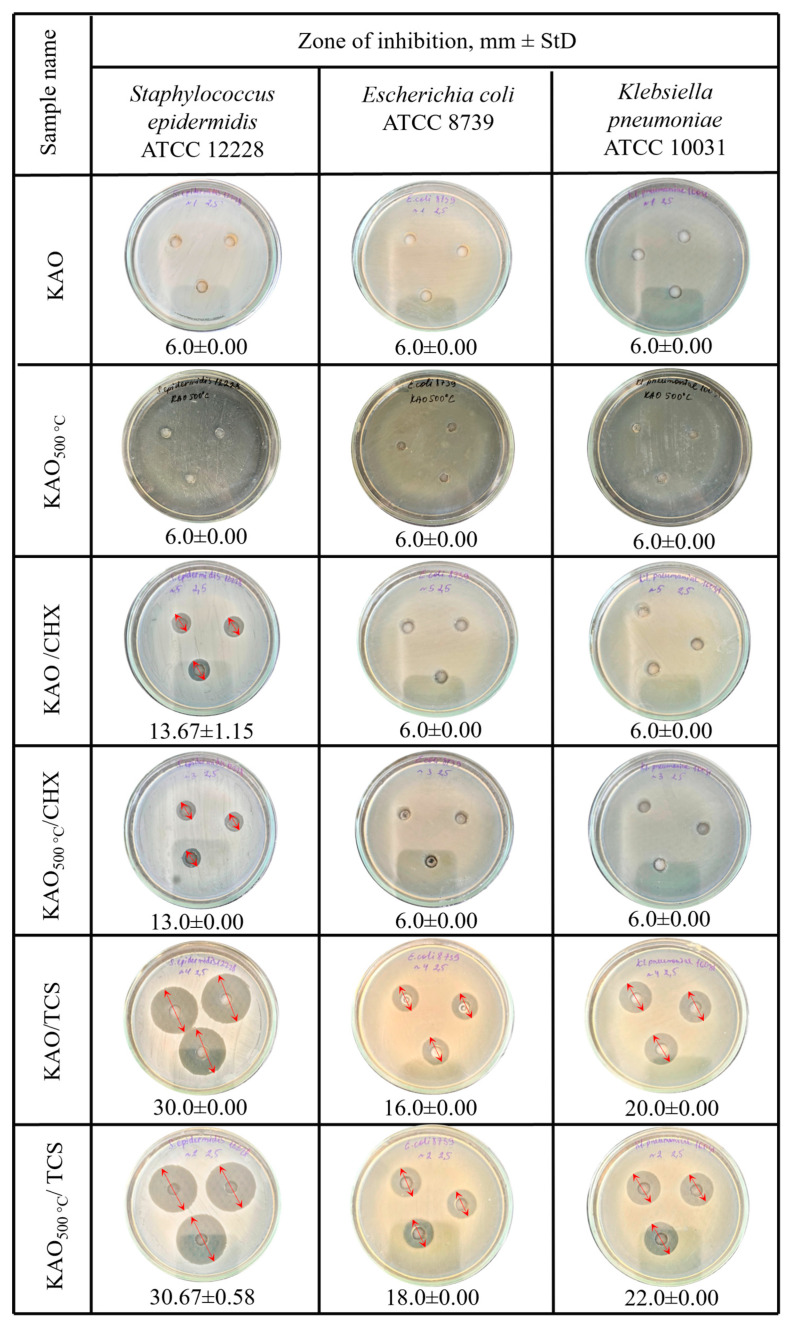
Results of the antimicrobial activity of natural kaolinite, calcined kaolinite, and composites kaolinite/CHX, kaolinite500 °C/CHX, kaolinite/TCS, and kaolinite500 °C/TCS on *Staphylococcus epidermidis* ATCC 12228, *Escherichia coli* ATCC 8739, and *Klebsiella pneumoniae* ATCC 10031 bacteria.

The antibacterial activity of natural kaolinite, KAO, KAO_500_, KAO/CHX, KAO_500_/CHX, KAO/TCS, and KAO_500_/TCS were tested via the well diffusion method against Gram-positive bacterium *Staphylococcus epidermidis* ATCC 12228 and Gram-negative bacteria *Klebsiella pneumoniae* ATCC 10031 and *Escherichia coli* ATCC 8739. The well diffusion test results are illustrated in Figure 7.

These bacterial species were selected for their common association with human infections [56], and the composites, having demonstrated antimicrobial potential against pathogens which can cause purulent wounds and diabetic foot, can help prevent wound infections [57].

Based on the antibacterial results (Figure 7), raw kaolinite (KAO) did not show any antibacterial activity. This once again confirmed that natural clay minerals themselves do not have antibacterial properties, unless specific antibacterial substances are adsorbed or incorporated on their surface [58]. The potential of the antibacterial activity of the kaolinite/chlorhexidine and kaolinite/triclosan composites, with respect to the Gram-positive bacterium *Staphylococcus epidermidis*, exhibited inhibition zones of 13 mm and 30 mm, respectively. For the Gram-negative bacterium *Escherichia coli*, the kaolinite/chlorhexidine and kaolinite/triclosan composites displayed 6 mm and 13 mm zones, respectively.

**Figure 8:**
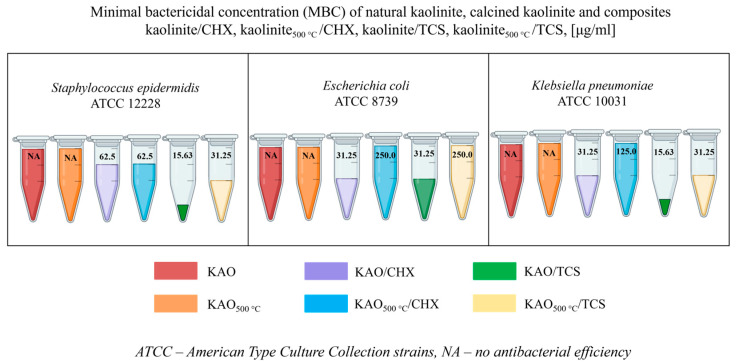
Results of the minimal bactericidal concentration of natural kaolinite, calcined kaolinite, and composites kaolinite/CHX, kaolinite500 °C/CHX, kaolinite/TCS, and kaolinite500 °C/TCS on *Staphylococcus epidermidis* ATCC 12228, *Escherichia coli* ATCC 8739, and *Klebsiella pneumoniae* ATCC 10031 bacteria.

The calcination of kaolinite at a higher temperature allows triclosan to be more effectively retained on the surface of kaolinite, which increases its effectiveness against bacteria. Calcination can create a more reactive surface capable of interacting with triclosan and bacteria, which enhances the antibacterial effect. In turn, for the composite containing chlorhexidine, the kaolinite-based composite calcined at 500 °C exhibited a similar inhibition zone of 13 mm as the non-calcined kaolinite. The minimum bactericidal concentration (Figure 8) of the kaolinite/TCS composite was 15.63 μg/mL for *Staphylococcus epidermidis* and *Klebsiella pneumoniae.*

*Staphylococcus epidermidis*, like many other Gram-positive bacteria, is sensitive to triclosan because it suppresses enzymes involved in the synthesis of fatty acids [59]. Comparing the antibacterial efficacies, samples containing triclosan show a better performance against Gram-positive and Gram-negative bacteria than samples without triclosan.

Chlorhexidine salts, which have a wide range of antimicrobial activity against Gram-positive and Gram-negative bacteria, are used to disinfect humans and animals [35]. Thus, all the obtained composites can become a promising basis for medical dressings.

Kaolinite modified with cetyltrimethylammonium bromide and copper has been confirmed to have a powerful antibacterial activity against *Pseudomonas aeruginosa* [60]. Moreover, kaolinite modification with cetylpyridinium bromide affected the total charge of kaolinite and its antibacterial activity against *Escherichia coli* [32]. Fe-porphyrin/kaolinite hybrid materials have been demonstrated as excellent antibacterial agents against the oral pathogens *Bacillus subtilis*, *Klebsiella pneumonia*, and *Escherichia coli* [61]. Nanocomposites including kaolinite have been also proposed as antibacterial materials [62]. The antibacterial activity of chlorhexidine-loaded silver–kaolinite has also been recently studied [35]. Chlorhexidine-impregnated montmorillonite–chitosan films have been tested as a potential wound dressing to prevent microbial colonization in wounds, and all films obtained have shown good antimicrobial activity [63]. Antibiotic triclosan was loaded into a chitosan–montmorillonite nanocomposite film, and a pH-sensitive sustained release was obtained. The high efficiency of film sterilization against *Staphylococcus aureus*, *Escherichia coli*, and *Staphylococcus epidermidis* has been established [64]. In [65], the antibacterial material chlorhexidine/vermiculite (CA/Ver) was successfully prepared using the intercalation process. All samples showed a very good antibacterial effect against *E. faecalis*, *S. aureus*, and *E. coli*, especially after 24 h or more [65].

The authors of [66] observed that the antibacterial activity of CA-Kaol is explained by the electrostatic attraction between the chlorhexidine cation and negatively charged bacterial cells. CA can adhere to the cell wall of a microorganism, violating the integrity of the cell membrane, which leads to cytoplasm leakage, membrane damage, enzyme inhibition, and ultimately, cell death [35].

## 4. Conclusions

Composites based on natural kaolinite and antibacterial agents chlorhexidine and triclosan were synthesized. SEM micrographs showed no changes in the structural or morphological characteristics of natural kaolinite, calcined kaolinite, and the chlorhexidine/kaolinite and triclosan/kaolinite composites. After impregnation with antibacterial agents, changes in the elemental composition of natural kaolinite were shown by SEM-EDX analysis. The appearance of peaks at 1474 and 1495 cm^−1^, indicated by FTIR spectroscopy, corresponded to the C-H bending vibrations of triclosan, and a 1500 cm^−1^ band was associated with chlorhexidine. X-ray diffraction analysis confirmed the presence of a structural disorder in the KAO_500°C_/CHX and KAO_500°C_/TCS composites, which is associated with the calcination of kaolinite. The antibacterial activities of the kaolinite/chlorhexidine and kaolinite/triclosan composites were investigated against Gram-positive *Staphylococcus epidermidis* and Gram-negative *Klebsiella pneumoniae* and *Escherichia coli* strains by the well diffusion method and dilution method. The highest zone of inhibition was observed against Staphylococcus epidermidis (30.00 ± 0.00 mm and 30.67 ± 0.58 mm) by applying KAO/TCS and KAO_500°C_/TCS via the well diffusion method. The minimum bactericidal concentration of the kaolinite/TCS composite was 15.63 μg/mL for *Staphylococcus epidermidis* and *Klebsiella pneumoniae*.

This study has a number of limitations that should be noted despite the promising results. First off, because biological systems are complicated, the antibacterial activity was assessed only in vitro, which might not fully represent the composites’ performance in vivo. Secondly, the study failed to evaluate the composites’ cytotoxicity or biocompatibility, which are critical for their use in pharmaceuticals. Furthermore, only a small number of bacterial strains were examined; a more thorough assessment of the antibacterial qualities would be possible by including a wider variety of microorganisms. Additionally, the calcination procedure was carried out at a single temperature, and the composites’ long-term stability and durability were not examined. The developed technology opens up a new way of modifying kaolinite-based composites via impregnation with the antibacterial agents chlorhexidine and triclosan in order to obtain new transdermal systems. Also, from this perspective, these composites can be used for the preparation of antibacterial dressings and ointments and as drug carriers.

## Data Availability

The original contributions presented in this study are included in the article. Further inquiries can be directed to the corresponding author.
